# Analysis of the efficacy and factors influencing survival of adjuvant radiotherapy for stage II-III biliary tract carcinoma

**DOI:** 10.1186/s12957-023-03209-0

**Published:** 2023-10-26

**Authors:** Yan-Ling Guo, Jia-Xing Guo, Jian-Guo Zhao, Ying-Na Bao

**Affiliations:** Department of Radiotherapy, The Affiliated Hospital of Inner Mongolia Medical University, No. 1 Tongdao North Street, Hohhot, 010050 China

**Keywords:** Biliary tract carcinoma, Treatment modality, Surgery, Adjuvant radiotherapy

## Abstract

**Background:**

To determine the efficacy of adjuvant radiotherapy for stage II–III biliary tract carcinoma.

**Methods:**

We retrospectively analyzed the data of 37 patients who underwent radical resection of biliary tract carcinomas at the Affiliated Hospital of Inner Mongolia Medical University between 2016 and 2020. We analyzed survival differences between patients who did (*n* = 17) and did not (*n* = 20) receive postoperative adjuvant radiotherapy by using Kaplan–Meier analysis. The log-rank test and Cox univariate analysis were used. The Cox proportional risk regression model was used for the multifactorial analysis of factors influencing prognosis.

**Results:**

The median survival time (28.9 *vs.* 14.5 months) and the 1-year (82.40% *vs.* 55.0%) and 2-year survival rates (58.8% *vs.* 25.0%) were significantly higher among patients who received adjuvant radiotherapy than among those who did not (*χ*^2^ = 6.381, *p* = 0.012). Multifactorial analysis showed that pathological tumor type (*p* = 0.004), disease stage (*p* = 0.021), and adjuvant radiotherapy (*p* = 0.001) were independent prognostic factors in biliary tract carcinoma. Subgroup analyses showed that compared to no radiotherapy, adjuvant radiotherapy significantly improved median survival time in patients with stage III disease (21.6 *vs.* 12.7 months; *p* = 0.017), positive margins (28.9 *vs.* 10.5 months; *p* = 0.012), and T3 or T4 tumors (26.8 *vs.* 16.8 months; *p* = 0.037).

**Conclusion:**

Adjuvant radiotherapy significantly improved the survival of patients with biliary tract carcinoma, and is recommended especially for patients with stage III disease, positive surgical margins, or ≥ T3.

## Highlights

1. Adjuvant radiotherapy was an independent prognostic factor for stage II–III BTC.

2. Adjuvant radiotherapy improved the survival of patients with stage III BTC.

## Background

Biliary tract carcinoma is a malignant tumor originating from the epithelial cells of the biliary system, and includes gallbladder carcinoma and cholangiocarcinoma. Cholangiocarcinomas are subdivided into intrahepatic, hilar, and distal extrahepatic cholangiocarcinomas, according to their anatomical location. Cholangiocarcinoma is the second most common malignant tumor of the liver after hepatocellular carcinoma, accounting for less than 1% of all human cancers [1]. Biliary tract carcinoma is highly aggressive and malignant, with a poor prognosis and a 5-year survival rate of less than 20% [2]. Currently, surgical resection is the mainstay of treatment for biliary tract carcinoma, but the high recurrence rate and low survival rate after surgery mean that adjuvant chemotherapy and radiotherapy are required to improve patient survival [3]. In recent years, researchers [4–6] have focused on the necessity and efficacy of adjuvant radiotherapy for biliary tract carcinoma. In the present study, we retrospectively reviewed the data of 37 postoperative patients with biliary tract carcinoma from our hospital in order to (i) determine whether postoperative adjuvant radiotherapy was associated with improvements in survival time and survival rate, and (ii) identify the factors affecting patient prognosis and survival after radical surgery for biliary tract carcinoma.

## Methods

### Patient selection

We retrospectively reviewed the clinical data of 37 patients who were diagnosed with biliary tract carcinoma and underwent radical surgery with or without adjuvant radiotherapy in the Affiliated Hospital of Inner Mongolia Medical University between January 2016 and December 2020. The inclusion criteria were as follows: clear pathological diagnosis showing that the primary tumor was biliary tract carcinoma, namely gallbladder cancer, hilar cholangiocarcinoma, intrahepatic cholangiocarcinoma, or extrahepatic cholangiocarcinoma; stage II–III disease; radical surgical resection; adjuvant chemotherapy and/or adjuvant radiotherapy; performance status score ≤ 1; complete treatment information; and clear follow-up time and follow-up results. The exclusion criteria were distant metastasis; preoperative or intraoperative adjuvant radiotherapy; presence of other malignant tumors; and patients who were lost to follow-up at an early stage and those who died from non-tumor causes.

### Treatment modalities

Patients with biliary tract carcinoma were treated with radical surgery, including cholecystectomy, cholecystectomy + regional lymph node dissection, cholecystectomy + local bile duct resection + regional lymph node dissection, hepatic segment or lobe resection + regional lymph node dissection, and pancreaticoduodenectomy. Postoperative adjuvant radiotherapy was administered to the surgical bed of the gallbladder and the regional lymph node drainage area, which included the hepatic hilar, pancreaticoduodenal, and abdominal trunk lymph nodes. The radiotherapy techniques used were 6 MV X-ray radiation administered via intensity-modulated radiotherapy (13 patients), three-dimensional conformal radiation therapy (3 patients), and spiral tomotherapy (1 patient). The doses ranged from 1.8 to 2.14 Gy/treatment session, 1 session/day, 5 sessions/week, for a total dose of 50–59.92 Gy and a median dose of 54 Gy. The limits for organs at risk were as follows: liver, V60 < 30 Gy; gastroduodenum, V50 < 5%; spinal cord, D_max_ < 40 Gy; left kidney, V20 < 20%; right kidney, V20 < 20%; and bladder, V50 < 50%.

Follow-up assessments were performed via telephone calls and outpatient clinic visits.

### Statistical analysis

Statistical analysis was performed using SPSS version 23.0 software. The final survival status of the patients was recorded with death as the endpoint event. For patients who were still alive by the end of the observation period, survival data were truncated at the end of 2021. The patients’ survival time was calculated as the interval from diagnosis to death or the truncation time. The median survival time, and 1- and 2-year survival rates were used as prognostic indicators. The Fisher exact probability method was used to compare count data, and the Kaplan–Meier method was used to perform survival analysis and plot survival curves. The log-rank test was used to compare survival rates, and the Cox proportional risk model was used to assess the relationship between treatment modalities and the risk of death on a univariate and multifactorial basis. A *p*-value of < 0.05 was considered to indicate a statistically significant difference.

### Theory/calculation

We collected the clinical data of 37 patients with biliary tract carcinoma after they had undergone surgical resection. The patients were divided into a postoperative adjuvant radiotherapy group and a no radiotherapy group. The median survival time and 1- and 2-year survival rates were analyzed with the Kaplan–Meier method. The following data were collected from all the patients: gender, age, tumor location, pathological tumor type, degree of tumor differentiation, incisional margin status, cholangiocarcinoma embolism, nerve invasion, lymph node metastasis, TNM stage (AJCC staging, 8th edition), and treatment modality. The Cox univariate and multivariate analyses were used to determine the independent prognostic factors in patients with biliary tract carcinoma, and the log-rank test was used to determine the association between survival time and each clinical feature. To further investigate the efficacy of adjuvant radiotherapy in postoperative patients with biliary tract carcinoma, we performed a subgroup analysis of incisional margin status, lymph node metastasis, TNM stage. The Kaplan–Meier method was used to evaluate the median survival time and 1- and 2-year survival rates in order to analyze the survival differences between the no-radiotherapy and adjuvant-radiotherapy groups.

## Results

### Clinical characteristics

A total of 37 patients were included in this study. They consisted of 15 men and 22 women. Their ages ranged from 43 to 88 years (median: 67 years). In all, 15 patients were diagnosed with gallbladder cancer, and 22 with bile duct cancer. According to AJCC staging (8^th^ edition), 8 patients had stage II disease, and 29 patients had stage III disease. All patients underwent surgery. Chemotherapy was performed for 25 patients, which was not synchronized with radiotherapy. Postoperative adjuvant radiotherapy was performed for 17 patients. We divided the patients into two groups according to the treatment modalities they received. The no-radiotherapy group included 20 patients (10 men and 10 women) aged 49–88 years (median: 68.5 years). The adjuvant-radiotherapy group consisted of 17 patients (5 men and 12 women) aged 43–70 years (median: 65 years). A detailed comparison of the clinical characteristics of the patients in the two study groups is shown in Table 1.

**Table 1:** Clinicopathological characteristics of patients with biliary tract carcinoma

**Factor**	**Treatment modality**		***P* value**
	No radiotherapy (*n* = 20)	Adjuvant radiotherapy (*n* = 17)	
Gender			0.204
Male	10	5	
Female	10	12	
Age (years)			1.000
≤ 65	9	9	
> 65	11	8	
Tumor location			1.000
Gallbladder cancer	9	6	
Cholangiocarcinoma	11	11	
Pathological tumor type			0.608
Adenocarcinoma	20	14	
Squamous cell carcinoma	0	3	
Tumor differentiation			0.701
Poor	7	3	
Moderate	7	11	
Well	6	3	
Surgical margins			0.408
R0	12	10	
R +	8	7	
Nerve invasion			0.202
Absent	10	11	
Present	10	6	
Vascular tumor thrombus			0.155
Absent	14	13	
Present	6	4	
Lymph node metastasis			1.000
N0	9	10	
N +	11	7	
T category			0.308
T2	6	2	
T3 or T4	14	15	
AJCC stage (8^th^ edition)			0.156
II	5	3	
III	15	14	
Adjuvant chemotherapy			0.389
Yes	11	14	
No	9	3	

### Univariate and multifactorial analyses

The total follow-up duration was 3.6–51.1 months after the surgery, and the median follow-up duration was 45.2 months. Of the 37 patients with biliary tract carcinoma, 30 died and 7 survived. Overall, the median survival time was 18.3 months, and the 1-year and 2-year survival rates were 70.3% and 40.4%, respectively. In the univariate analysis, the following factors were found to influence the postoperative prognosis of patients with biliary tract carcinoma: pathological tumor type (*p* = 0.009), vascular tumor thrombus (*p* = 0.027), tumor stage (*p* = 0.034), and treatment modality (*p* = 0.012; Table 2). Multifactorial analysis of the statistically significant factors showed that pathological tumor type (*p* = 0.004), tumor stage (*p* = 0.021), and treatment modality (*p* = 0.001) were independent prognostic factors for patients with biliary tract carcinoma. Gender, age, tumor differentiation, cholangiocarcinoma embolism, surgical margin status, lymph node metastasis, and T-stage were not significantly correlated with prognosis (Table 3).

**Table 2:** Univariate analysis of prognostic factors for patients with biliary tract carcinoma

**Factor**	**Univariate analysis**	
	**Median survival (months)**	***P* value**
Gender		0.365
Male	16.7	
Female	18.4	
Age (years)		0.459
≤ 65	18.4	
> 65	16.7	
Tumor location		0.540
Gallbladder cancer	17.1	
Cholangiocarcinoma	18.4	
Pathological type		0.009
Adenocarcinoma	9.7	
Squamous cell carcinoma	18.4	
Tumor differentiation		0.169
Poor	14.5	
Moderate	24.3	
Well	18.4	
Surgical margin		0.664
R0	17.1	
R +	18.4	
Nerve invasion		0.134
Absent	16.8	
Present	24.1	
Vascular tumor thrombus		0.027
Absent	24.1	
Present	13.8	
Lymph node metastasis		0.185
N0	24.1	
N +	17.1	
T category		0.362
T2	10.5	
T3 or T4	18.3	
AJCC stage (8^th^ edition)		0.034
II	26.4	
III	16.8	
Treatment modality		0.012
No radiotherapy	14.5	
Adjuvant radiotherapy	28.9

**Table 3:** Multivariate analyses of prognostic factors for patients with biliary tract carcinoma

**Factor**	**Hazard ratio**	**95% CI**	***P* value**
Pathological type	0.092	0.018–0.476	0.004
Vascular tumor thrombus	1.013	0.397–2.587	0.978
AJCC stage (8^th^ edition)	3.460	1.209–9.899	0.021
Treatment modality	0.204	0.079–0.527	0.001

*CI* Confidence interval

According to the grading standards of the Radiation Therapy Oncology Group, 4 (23.53%) patients in the radiotherapy group developed grade I–II gastrointestinal reactions after the treatment, which mainly included decreased appetite, and nausea and vomiting. Bone marrow suppression and abdominal pain occurred in 3 (17.65%) patients each.

### Survival analysis

**Fig. 1:**
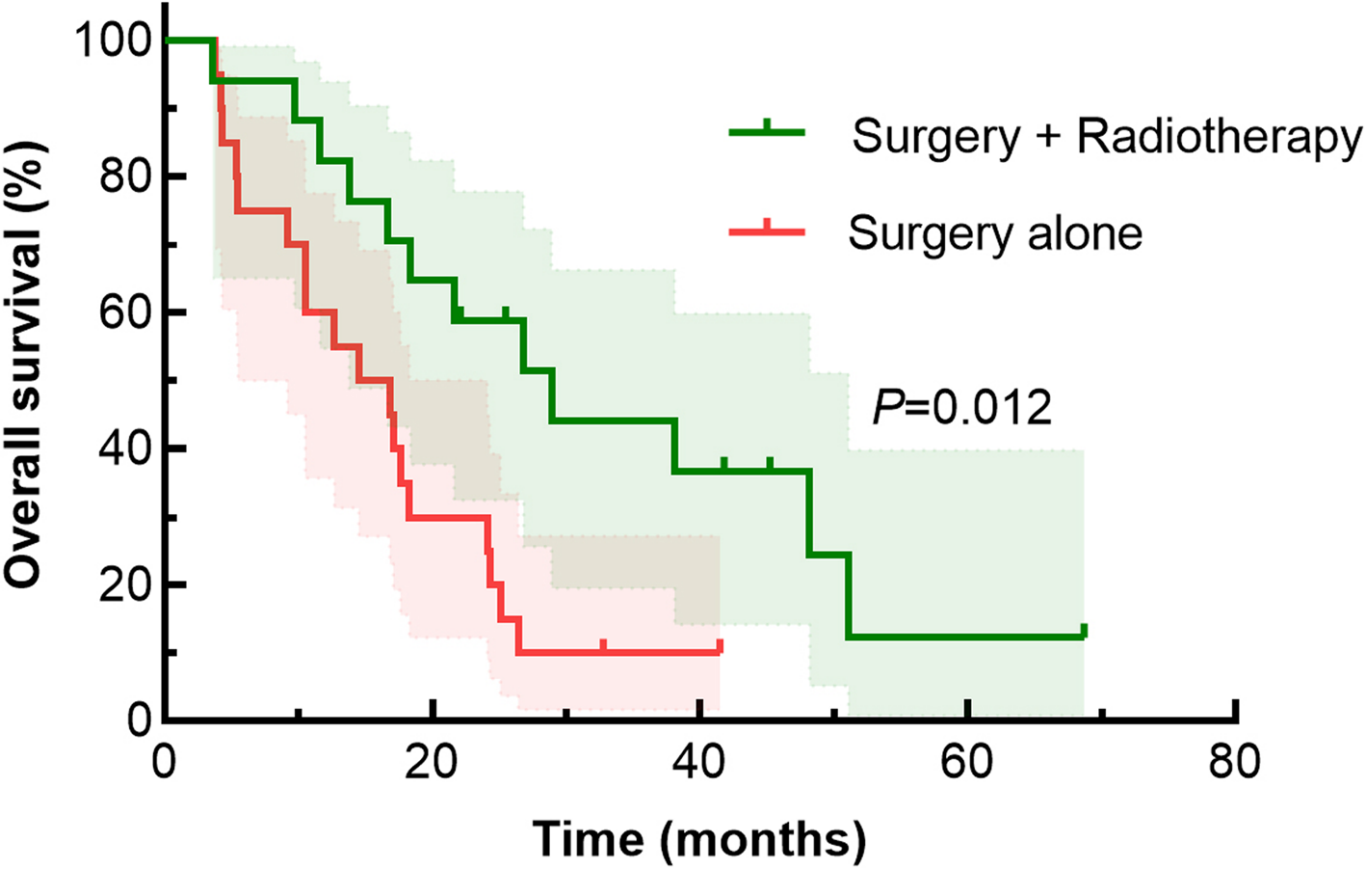
Comparison of Kaplan–Meier survival curves of patients with biliary tract carcinoma treated with different therapeutic modalities

The Kaplan–Meier method was used for the survival analysis of the patients in the no-radiotherapy and adjuvant-radiotherapy groups, and the survival curves of the two groups were plotted (Fig. 1). The median survival time in the no-radiotherapy group was 14.5 months, and the survival rates at 1 and 2 years were 55.0% and 25.0%, respectively. The median survival time in the adjuvant-radiotherapy group was 28.9 months, and the survival rates at 1 and 2 years were 82.40% and 58.8%, respectively. The difference between the two groups was statistically significant (*χ*^*2*^ = 6.381, *p* = 0.012).

**Fig. 2:**
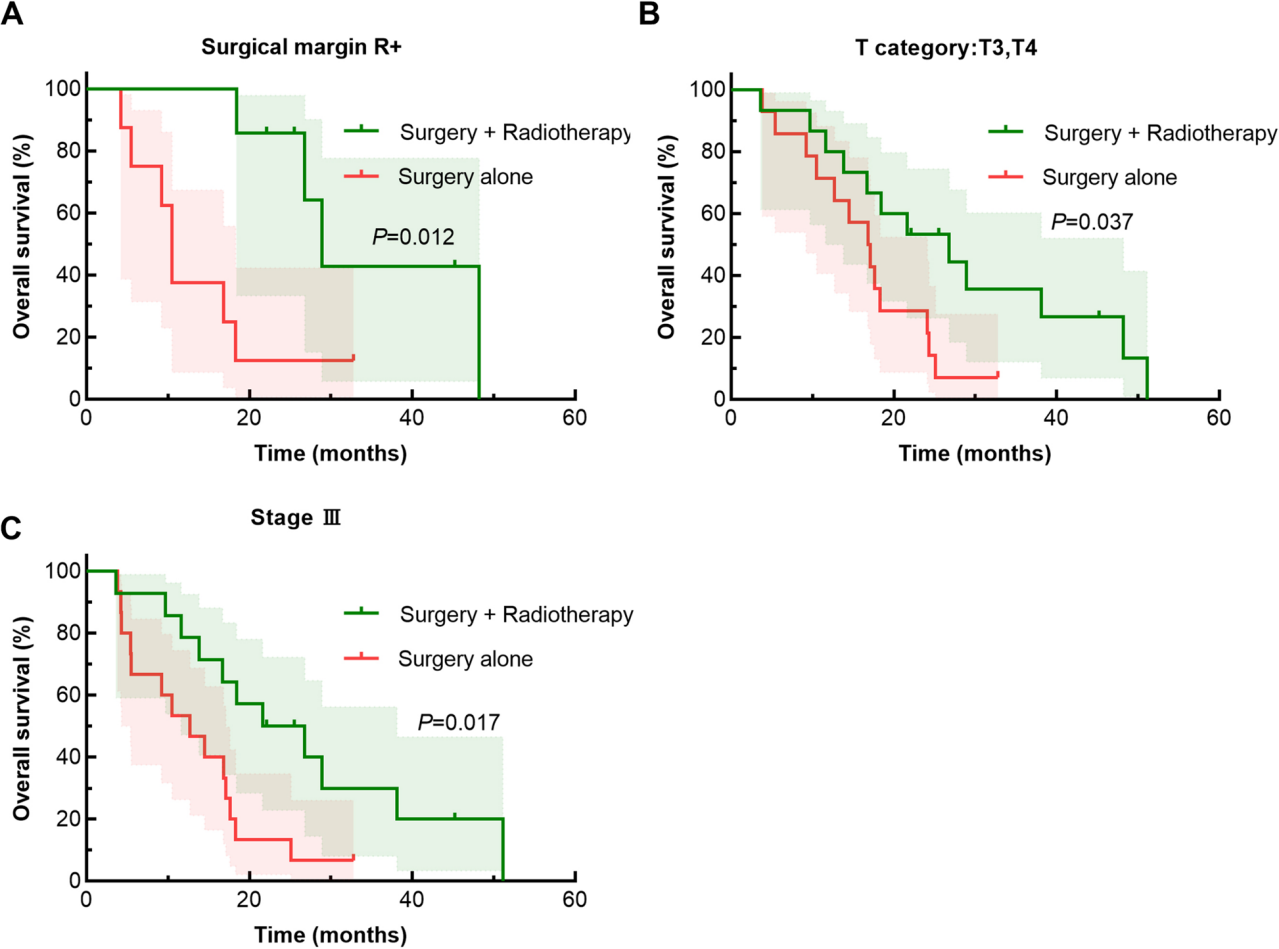
Subgroup analyses of Kaplan–Meier survival curves of patients with biliary tract carcinoma. **A** Positive surgical margins, **B** T3 or T4 tumors, and **C** stage III disease

We next performed subgroup analyses based on clinicopathological characteristics such as TNM stage, surgical margin status, and lymph node metastasis to further analyze the survival differences between the no-radiotherapy and adjuvant-radiotherapy groups (Table 4). The results showed no significant differences in survival time between the two treatment modalities within the R0 (*p* = 0.355), T2 (*p* = 0.092), stage II (*p* = 0.053), N0 (*p* = 0.089), and N + subgroups (*p* = 0.133). In contrast, significant differences in survival time between the no-radiotherapy group and adjuvant radiotherapy groups were found within the R + , T3/T4, and stage III subgroups (Fig. 2). Among patients with positive surgical margins (R + subgroup), adjuvant radiotherapy significantly improved the median survival time (28.9 *vs.* 10.5 months; *p* = 0.012). Among patients with T3 or T4 tumors, adjuvant radiotherapy significantly improved the median survival time (26.8 *vs.* 16.8 months) and the survival rate at 2 years (53.3% *vs.* 21.4%, *p* = 0.037). Finally, among patients with stage III disease, the adjuvant radiotherapy significantly improved both median survival time (21.6 *vs.* 12.7 months) and 1- and 2-year survival rates (78.6% and 40.0% *vs.* 46.7% and 6.7%, respectively; *p* = 0.017).

**Table 4:** Subgroup analyses of median survival time and survival rates among patients with different treatment modalities

**Factor**	**Treatment modality**	**No**	**Median survival** **(months)**	**1-year survival rate**	**2-year survival rate**	***P* value**
R0	No radiotherapy	12	17.1	66.7%	33.3%	0.355
	Adjuvant radiotherapy	10	16.7	70.0%	40.0%	
R +	No radiotherapy	8	10.5	37.5%	12.5%	0.012
	Adjuvant radiotherapy	7	28.9	85.7%	64.3%	
T2	No radiotherapy	6	——	33.3%	16.7%	0.092
	Adjuvant radiotherapy	2	——	——	——	
T3 or T4	No radiotherapy	14	16.8	64.3%	21.4%	0.037
	Adjuvant radiotherapy	15	26.8	80.0%	53.3%	
Stage II	No radiotherapy	5	24.3	80.0%	40.0%	0.053
	Adjuvant radiotherapy	3	48.2	——	——	
Stage III	No radiotherapy	15	12.7	46.7%	6.7%	0.017
	Adjuvant radiotherapy	14	21.6	78.6%	40.0%	
N0	No radiotherapy	9	16.8	55.6%	33.33%	0.089
	Adjuvant radiotherapy	10	38.1	80.0%	60.0%	
N +	No radiotherapy	11	14.5	54.5%	9.1%	0.133
	Adjuvant radiotherapy	7	26.8	85.5%	28.6%

## Discussion

### Challenges in improving the prognosis of BTC solely through surgery

The present study identified pathological tumor type, postoperative adjuvant radiotherapy, and tumor stage as independent prognostic factors for patients with biliary tract carcinoma, and showed that adjuvant radiotherapy after radical resection could effectively improve the prognosis of patients (hazard ratio [HR] = 0.204, *p* = 0.001). Among patients with biliary tract carcinoma, a common cause of death is local biliary obstruction leading to liver failure or biliary sepsis [7]. At present, radical surgery is the primary treatment for patients with biliary tract carcinoma and is performed as soon as possible after diagnosis [8]. However, even after surgical treatment, the 5-year overall survival (OS) rates of these patients are only 27% to 37% [2]. Due to the close anatomical relationship between the tumor and the hepatic hilum and the surrounding tissues and organs, and the abundance of local lymphatic tissues, biliary tract carcinoma is often associated with local infiltration and lymph node metastasis, which complicates surgery [9, 10]. Local recurrence is extremely common after radical surgery, with reported recurrence rates of > 50% and a mean time to recurrence of 10–20 months [11, 12]. The high recurrence rate after surgery for biliary tract carcinoma makes improving patient prognosis a major challenge.

Wang et al. [13] reported that local extrahepatic metastasis, vascular infiltration, lymph node metastasis, tumor diameter > 5.5 cm, carcinoembryonic antigen level > 2.5 μg/L, and CA19-9 level > 41.2 U/mL were independent risk factors for postoperative recurrence and death in patients with intrahepatic cholangiocarcinoma. The authors identified these risk factors by establishing a nomogram, which predicted the prognosis of patients with intrahepatic cholangiocarcinoma more accurately than did five other intrahepatic cholangiocarcinoma staging systems that were used at that time. Song et al. [14] retrospectively analyzed the OS and local recurrence rates of 115 patients with intrahepatic cholangiocarcinoma after surgery; the authors found that the 5-year OS and local control rates were 49.1% and 51.6%, respectively, and the site of local recurrence was most commonly in the liver and peritoneum.

Yoshiaki et al. [15] reported that the 5-year survival rates of 127 postoperative patients with intrahepatic, hilar, and distal cholangiocarcinoma were 37%, 37%, and 43%, respectively; the authors concluded that tumor location was not significantly correlated with survival among patients with cholangiocarcinoma. In the present study, the clinicopathological data of 37 patients with biliary tract carcinoma were retrospectively analyzed. Patients with intrahepatic, hilar, and extrahepatic bile duct carcinoma were unified into a single cholangiocarcinoma group, which included 15 patients with gallbladder cancer and 22 patients with cholangiocarcinoma. Univariate and multifactorial analyses showed that pathological tumor type, postoperative adjuvant radiotherapy, and tumor stage were independent prognostic factors for patients with biliary tract carcinoma, while tumor location, lymph node metastasis, and postoperative margin status were not significantly correlated with prognosis. Surprisingly, we found that adjuvant radiotherapy could effectively improve patient prognosis (HR = 0.204, *p* = 0.001). These findings show that postoperative local control remains important in improving patient prognosis.

Radiation therapy is currently considered an effective local treatment for potential microscopic lesions of biliary tract carcinoma, and can delay local postoperative recurrence and disease progression through local regional treatment [16]. However, few studies have evaluated the role of adjuvant radiotherapy in the treatment of biliary tract carcinoma, and the choice of whether or not to offer adjuvant radiotherapy and radiotherapy for biliary tract carcinoma remains controversial [17]. Our study found that adjuvant radiotherapy significantly prolonged the median survival time of patients with biliary tract carcinoma as compared with the no-radiotherapy group (14.5 *vs.* 28.9 months, *p* = 0.012) and improved the 1- and 2-year survival rates of the patients.

### Value of adjuvant radiotherapy in the treatment of BTC

However, Horgan et al. [18] conducted a meta-analysis of 6712 patients with malignancies of the biliary system treated between 1960 and 2010, and found no significant improvement in OS in the adjuvant radiotherapy and chemoradiotherapy groups as compared with the resection alone group (OR = 0.74, *p* = 0.06). Liang et al. [19] and Glazer et al. [20] also reported that neither neoadjuvant radiotherapy nor adjuvant radiotherapy improved the OS of patients with biliary malignancies, and that ensuring a negative surgical margin of 1 cm or more was the best way to enable long-term patient survival.

Beltran et al. [21] found that adjuvant radiotherapy significantly improved OS in patients with extrahepatic bile duct cancer (HR = 0.62, 95% confidence interval [CI]: 0.48 to 0.78, *p* < 0.001). Choi et al. [22] conducted a meta-analysis on adjuvant radiotherapy for extrahepatic cholangiocarcinoma, and included the enrolled studies in a sensitivity analysis to minimize selection bias and increase the accuracy of the study. The results showed that 5-year survival rates were 27.8% and 34.5% (*p* = 0.11) in the surgery-alone and adjuvant-radiotherapy groups, respectively, with local recurrence rates of 52.1% and 34.9% (*p* = 0.014), respectively. The above findings indicate that adjuvant radiotherapy should be actively encouraged in postoperative patients with cholangiocarcinoma. To further explore the value of adjuvant radiotherapy in biliary tract carcinoma, Wang et al. [23] developed a survival prediction model based on the SEER data of 4,180 postoperative gallbladder cancer patients; the authors found that adjuvant radiotherapy offered a significant survival advantage for postoperative patients with pT ≥ T2. Given that in patients with advanced T stage and lymph node metastasis, complete radical resection may be difficult to achieve due to extensive lesion infiltration, adjuvant radiotherapy can effectively reduce the recurrence of microscopic lesions. In the retrospective studies conducted by Kim et al. and Ren et al., adjuvant radiotherapy was found to be more beneficial in lymph node-positive patients than in lymph node-negative patients (HR = 0.54), and significantly reduced the risk of death and recurrence compared with the surgery-only group (HR = 0.61) [24, 25].

Giving adjuvant radiotherapy to patients with gallbladder cancer was reported to be a significant predictor of improved OS [26]. A retrospective analysis of nomograms constructed based on SEER and Medicare data showed that radiotherapy was more beneficial than chemotherapy alone in improving patient prognosis for all patients, with a significant benefit especially for patients with ≥ T2 and positive lymph nodes [27]. In a prospective single-arm trial of chemotherapy combined with radiotherapy (SWOG0809) [28], a total of 79 patients with a specific pathological stage of T2–4 or N1 were enrolled, of whom 69 patients received concurrent chemoradiotherapy with capecitabine (1,330 mg/m^2^ twice daily, 7 days a week) and a radiotherapy regimen of 45 Gy to the local lymph nodes and 54–59.4 Gy to the preoperative tumor bed. The 2-year survival rate was 65% (95% CI: 53%–74%) for all patients, and 67% and 60% for R0 and R1 patients, respectively. These results suggested that chemotherapy combined with radiotherapy is an effective adjuvant treatment for patients with biliary tract carcinoma. This study was a phase II clinical trial with a high level of evidence, and the high quality of the study protocol greatly improved trial homogeneity and reduced variability, and the results will be of great interest in future treatment and research of biliary tract carcinoma.

As biliary tract carcinoma is relatively rare in clinical practice. Currently, few high-quality studies with useful data are available to guide clinical practice, and clinicians’ decisions are mostly based on clinical guidelines and treatment experience. The National Comprehensive Cancer Network guidelines for gallbladder and bile duct cancers recommend adjuvant radiotherapy for patients with positive margins or definite lymph node metastases [29]. Based on prospective clinical findings of malignancies of the biliary system [28], the American Society of Clinical Oncology issued clinical practice guidelines in 2019 [30]. They recommend radiotherapy for patients with R1 resection of hilar cholangiocarcinoma, for patients with extrahepatic cholangiocarcinoma or gallbladder cancer, and for patients with microscopically positive surgical margins. They endorse a target area dose of 54–59.4 Gy in the postoperative tumor bed and 45 Gy in the regional lymph node drainage area.

In our study, adjuvant radiotherapy and staging were independent prognostic factors for postoperative patients with biliary tract carcinoma, and adjuvant radiotherapy was effective in prolonging survival times and increasing survival rates. Moreover, adjuvant radiotherapy is beneficial for patients with biliary tract carcinoma, and it should be recommended for patients with high risk factors for recurrence, such as positive surgical margins, ≥ T3, and stage III disease.

The complications of postoperative radiotherapy mainly include gastrointestinal reactions. Mild adverse events include nausea, vomiting, and diarrhea; severe radiation injuries include gastrointestinal ulcer, biliary stricture, intestinal obstruction, and radiation hepatitis, which can result in severe abdominal pain and gastrointestinal bleeding, and can even be life-threatening [31, 32]. However, with advances in radiotherapy technology, the improved protection of the organs at risk has greatly reduced the occurrence of radiotherapy-related adverse reactions. In our study, the incidence of the side effects of radiotherapy was acceptable, and these mainly included grade I–II myelosuppression and grade I–II gastrointestinal reactions.

The present study was a single-center retrospective study with a small sample size. Only patients with complete case data and follow-up results who met the inclusion criteria were included in the study, and patients who were lost to follow-up were excluded. This may have led to a selection bias. Patients with local recurrence and distant metastasis were not specifically included in the study. Several of the retrospective studies cited above have shown that adjuvant radiotherapy is an important prognostic factor in lymph node-positive patients, but the present study did not demonstrate a benefit of adjuvant radiotherapy in lymph node-positive patients by univariate, multifactorial, or subgroup analyses. Further analysis with a larger sample size is needed.

## Conclusions

The incidence of biliary tract carcinoma is low in clinical practice, and most current studies are retrospective in nature; therefore, there is insufficient evidence for the survival benefit of adjuvant radiotherapy after surgery for biliary tract carcinoma. This study provides a reference for a large-scale prospective clinical study and for the clinical application of adjuvant radiotherapy after surgery for biliary tract carcinoma. The study also provides supportive evidence for the continued exploration of the potential benefits of adjuvant radiotherapy after surgery for biliary tract carcinoma.

## Data Availability

The datasets generated and analyzed during the present study are available from the corresponding author on reasonable request.

## Abbreviation

BTC: Biliary tract carcinoma
